# Differentiation and Glucocorticoid Regulated Apopto-Phagocytic Gene Expression Patterns in Human Macrophages. Role of Mertk in Enhanced Phagocytosis

**DOI:** 10.1371/journal.pone.0021349

**Published:** 2011-06-24

**Authors:** Gábor Zahuczky, Endre Kristóf, Gyöngyike Majai, László Fésüs

**Affiliations:** 1 Apoptosis and Genomics Research Group, Department of Biochemistry and Molecular Biology, Hungarian Academy of Sciences, University of Debrecen, Debrecen, Hungary; 2 Third Department of Internal Medicine, University of Debrecen, Debrecen, Hungary; University of North Dakota, United States of America

## Abstract

The daily clearance of physiologically dying cells is performed safely mainly by cells in the mononuclear phagocyte system. They can recognize and engulf dying cells utilizing several cooperative mechanisms. In our study we show that the expression of a broad range of apopto-phagocytic genes is strongly up-regulated during differentiation of human monocytes to macrophages with different donor variability. The glucocorticoid dexamethasone has a profound effect on this process by selectively up-regulating six genes and down-regulating several others. The key role of the up-regulated mer tyrosine kinase (Mertk) in dexamethasone induced enhancement of phagocytosis could be demonstrated in human monocyte derived macrophages by gene silencing as well as blocking antibodies, and also in a monocyte-macrophage like cell line. However, the additional role of other glucocorticoid induced elements must be also considered since the presence of autologous serum during phagocytosis could almost completely compensate for the blocked function of Mertk.

## Introduction

The efficient elimination of apoptotic cells or those dying through necrosis is performed mainly by the cells of the mononuclear phagocyte system [1]–[2]. Circulating monocytes, resident macrophages and those that infiltrate tissues or divide locally in circumstances of injury or inflammation are the major elements of this system [3]. The process of apoptotic cell corpse removal by professional phagocytes is remarkably complex and only partly defined [4]–[6]. It consists of two major steps: (1) recognition and (2) subsequent engulfment of apoptotic cells [1]. Ligands appearing on the apoptotic cells, receptors on the phagocyte and bridging molecules in the environment may act to drive either or both of these steps [7], [33]. While elements of the recognition and receptor elements of the apopto-phagocytic machinery seem to be highly redundant [8], the signaling pathways for the engulfing machinery converge to switch on rac-1 dependent cytoskeletal processes [7].

Glucocorticoids (GC) have an extensive range of effects in target tissues throughout the organism eliciting both rapid and delayed changes in physiological functions and pathologic tissues environment. Their therapeutic effects are mediated by the classical cytosolic glucocorticoid receptors (cGCRs) which move to the nucleus to regulate gene expression following ligand binding or by membrane-bound GCR and direct interactions with the cell membrane [9]–[10]. The potentiating effect of glucocorticoids on the phagocytosis of apoptotic neutrophils, which can be inhibited by GCR antagonists, has been described [11]–[12]. As an explanation of the enhanced phagocytic uptake of apoptotic cells, an increased capacity for engulfment oriented reorganization of cytoskeletal elements, loss of phosphorylation of adhesion mediators (paxillin and pyk2) and increased amount of Rac GTPase were considered [13]–[14]. By analyzing the GC-induced expression patterns in human monocytes by microarray technology the following pathways and gene-clusters were proposed as possible functional markers of the developing anti-inflammatory subtype: up-regulated antioxidative, migration/chemotaxis, phagocytosis, anti-inflammatory genes and down-regulated T-cell chemotaxis, adhesion, apoptosis, oxidative functions and IFNγ regulated genes. [15].

The importance of Mer tyrosine kinase (Mertk), as a member of of the Tyro3/Axl/Mer family of receptor tyrosine kinases in the engulfment and efficient clearance of apoptotic cells has been clearly demonstrated [16] and it was recently found that the glucocorticoid dexamethasone (DXM) treated human monocyte derived macrophages (HMDMs) exhibit augmented capacity of phagocytosis only in the presence of a serum factor that was identified as protein S, a ligand for Mertk. [17].

Here, we investigated the effects of differentiation and treatment by DXM on the gene-expression pattern of HMDMs using a custom designed apopto-phagocyte panel. Our data show that during differentiation of monocytes to macrophages most of the apopto-phagocytic genes are highly up-regulated. Dexamethasone led to further up-regulation of 6 genes while some others were significantly down-regulated. Of the up-regulated ones only silencing of Mertk could prevent DXM-mediated increase in phagocytosis of apoptotic cells in a serum-independent manner; this observation was confirmed by applying blocking antibodies against Mertk and showing that in monocytic cell lines low level and lack of Mertk inducibility by DXM is accompanied by their inability to engulf apoptotic cells.

## Materials and Methods

### Ethics Statement

Human monocytes were isolated from ‘buffy coats’ of healthy blood donors. Buffy coats were provided anonymously by the Hungarian National Blood Service where blood were taken from healthy volunteers and written informed consent from all participants were obtained. For these studies approval was obtained from the ethics committee of the Medical and Health Science Center, University of Debrecen (DEOEC RKEB/IKEB Prot. No. 2745 -2008). The ethics committee approved this consent procedure.

### Preparation of cells, apoptosis and phagocytosis quantification assays

Human monocytes from ‘buffy coats’ of healthy blood donors were isolated by Ficoll–Paque Plus (Amersham Biosciences, Uppsala, Sweden) gradient and magnetic separation using CD14 human microbeads (Miltenyi Biotec, Auburn, CA, USA). Human macrophages were obtained through a 5-day differentiation using 5 ng/ml macrophage colony-stimulating factor (M-CSF) (Peprotech EC, London, United Kingdom) at 37°C at a cell density of 1×10^6^ cells/ml in Iscove's Modified Dulbecco's Medium (IMDM) (Gibco) containing 10% human AB serum in a 5% CO_2_ atmosphere. Cytokines were added on day 0 and day 3. The glucocorticoid treated cells were differentiated in the presence of 1 µM DXM (Sigma–Aldrich St. Louis, USA). Neutrophil granulocytes were isolated by Histopaque (Sigma–Aldrich) fractionation of EDTA-treated venous blood following erythrocyte sedimentation using 3% filtered dextrane solution. Default apoptosis in neutrophils was obtained by culture of freshly isolated cells in IMDM containing 10% human AB serum for 20 h at 37°C in a 5% CO_2_ atmosphere. Cell death was assessed by the Annexin-V-fluorescein isothio-cyanate Apoptosis Detection Kit (MBL, Woburn, MA, USA) according to manufacturer's recommendations; proportion of stained Annexin-V^+^ and Annexin-V^+^ propidium iodide^+^ (PI^+^) cells was determined by fluorescence activated cell sorter (FACS) analysis on BD Bioscience flow cytometer. Mono Mac 6 and THP-1 cells were maintained in RPMI 1640 medium containing 10% Fetal Bovine Serum (FBS), l-glutamine (300 mg/l), penicillin and streptomycin (Sigma–Aldrich). The cells were washed with phosphate-buffered saline (PBS) three times, followed by incubation in 10% FCS-RPMI, 100 nM PMA for 72 h at 37°C at a cell density of 5×10^5^ cells/ml in a 5% CO_2_ atmosphere. For vitamin D3 and dexamethasone treated THP-1 cells, 0.1 µM DXM concentration was applied.

Dying neutrophils were labeled with carboxyfluoresceindiacetate-succinimidyl ester (CFDA-SE, Invitrogen, 15 µM, overnight), washed free of conditioned media and resuspended in PBS before their addition to a prewashed Cell Tracker Orange 5-(and-6)-(((4-chloromethyl) benzoyl)amino)tetramethylrhodamine labeled (CMTMR, Invitrogen, 3,75 µM, overnight) macrophage monolayer. Before adding dying neutrophils, macrophages were preincubated with anti-Mertk blocking antibodies for 10 min at a concentration of 10 µg/ml. Macrophages and apoptotic neutrophils were mixed at a ratio of 1∶5 and incubated for 30 min at 37°C in 5% CO_2_ atmosphere. The assay was performed in the presence or absence of human 10% AB serum. The whole-cell mixture was collected by trypsin digestion, centrifuging, washing twice in PBS and fixing in 1% PBS-buffered paraformaldehyde (pH 7.4). The net phagocytosis rate was determined by FACS analysis (BD Bioscience) as percent phagocytic cells that have engulfed (positive for both CMTMR and CFDA) as previously described [34].

### RNA preparation and TaqMan real-time RT-PCR

Total cellular RNA was isolated from untreated and dexamethasone treated human monocyte-derived macrophages using TRIzol Reagent (Invitrogen Life Technologies). Pre-designed, factory-loaded 384- well TaqMan low-density array (Applied Biosystems, Foster City, CA, USA) was used to determine the level of expression of genes listed in Table S1. Two replicates per target gene and two parallels per biological sample were carried out. Expression levels of target genes were normalized to 18S rRNA as endogenous control. Gene expression values were calculated based on the ΔΔC_t_ method, where one sample was designated as calibrator, through which all other samples were analyzed. ΔC_t_ represents the threshold cycle (C_t_) of the target minus that of 18S rRNA and ΔΔC_t_ represents the ΔC_t_ of each target minus that of the calibrator. Relative quantities (RQ or fold changes) were determined using the equation where relative quantity equals 2^−ΔΔCt^ (average of three repeated experiments).

### siRNAs and electroporation of human monocyte derived macrophages

In order to knock-down each investigated genes, siRNA constructs were obtained from Ambion, targeting adenosine A3 receptor [3′-CGUCUAUGCCUAUAAAAUAtt (sense) and 5′-UAUUUUAUAGGCAUAGACGat (antisense)], AXL receptor tyrosine kinase [3′-GGAACUGCAUGCUGAAUGAtt (sense) and 5′-UCAUUCAGCAUGCAGUUCCtg (antisense)], complement C1q subcomponent subunit A [3′-CCAACCAGGAAGAACCGUAtt (sense) and 5′- UACGGUUCUUCCUGGUUGGtt (antisense)], c-mer proto-oncogene tyrosine kinase [I: 3′- CAGUAGCCGUGUUAACGAAtt (sense) and 5′-UUCGUUAACACGGCUACUGtt (antisense); II: 3′-GAACUUACCUUACAUAGCUtt (sense) and 5′-AGCUAUGUAAGGUAAGUUCaa (antisense)] and thrombospondin-1 [3′-GGACUGCGUUGGUGAUGUAtt (sense) and 5′- UACAUCACCAACGCAGUCCtt (antisense)]. Non-targeting siRNA negative control (scrambled) was obtained from Sigma-Genosys (3781976-F/112). Three days after isolation HMDMs were harvested from the 24 well plates and washed once with IMDM and once with PBS (all at room temperature). The cells were resuspended in OptiMEM without phenol red (Invitrogen Life Technologies) at a concentration of 4×107/ml. siRNA was transferred to a 4-mm cuvette (3 µM final concentration or as indicated). A volume of 100 µl of cell suspension was added and incubated for 3 min before being pulsed in a Genepulser Xcell (Bio-Rad). Pulse conditions were square-wave pulse, 500 V, 0.5 ms. Immediately after electroporation, the cells were transferred to human AB serum supplemented IMDM with the previously indicated concentrations of M-CSF and DXM.

### Quantitative PCR analysis of the knock-down effect in HMDMs and THP-1 cells

Total cellular RNA was isolated from electroporated macrophages and THP-1 cells as previously described. Total RNA concentrations were quantified by spectrometry after DNase treatment (Sigma-Aldrich). TaqMan reverse transcription reagent kit (Applied Biosystems) was used for generating cDNA according to manufacturers instructions using 200 ng of total RNA in a 20 µl reaction volume. An ABI Prism 7700 sequence detection system (Applied Biosystems) was used to determine relative gene expression. Gene primers and probes were designed and supplied by Applied Biosystems. Human cyclophyllin was used as endogenous control to normalize the amount of the sample cDNA added to the reaction. The human cyclophyllin primers were labelled with TAMRA and sample primer with FAM. All samples were run in triplicate. Relative mRNA expression was quantified by comparing the cycle threshold (Ct) between control and knock-down cell samples.

### Antibodies and immunoblotting

Mouse monoclonal antibodies against Mertk were purchased from R&D systems (Minneapolis, USA) (MAB8911) and ABCAM (ab52591) (Cambridge, MA, USA).

Human macrophages were collected and washed with PBS followed by lysing in 50 mMTris–HCl; 0.1% Triton X-100; 1 mM EDTA; 15 mM 2-MEA and proteinase inhibitors. Insoluble cellular material was removed by centrifugation and the lysates were mixed with 5× Laemmli loading buffer (LB), boiled for 10 min and loaded onto a 10% SDS polyacrylamide gel. Proteins were transferred onto PVDF membranes followed by blocking with 5% skimmed milk. Membranes were probed by monoclonal anti-Mertk antibody (ab52591) and β-actin (Sigma-Aldrich) overnight at 4°C, followed by incubation with horseradish-peroxidase (HRP)-conjugated anti-mouse antibody (Sigma-Aldrich) for 1 h at room temperature. Immunoblots were developed with Immobilon Western chemiluminescent substrate (Millipore).

### Statistical analysis

Statistical analysis of phagocytosis data was performed by using the paired Student's t-test (two tailed). Statistical evaluation of the expression changes was performed in “R” using BioConductor implementing a moderate t-test based on Bayesian statistic which allows for a comparably reliable estimation of the SD even in case of few biological replicates. Correlation between gene expression levels and phagocytosis was evaluated using linear regression by *Partial Least Squares (PLS)* module of Statistica software that is a comprehensive implementation of partial least squares regression analysis.

## Results

### Induction of apopto-phagocytic genes during differentiation of monocytes to macrophages

During differentiation of monocytes to macrophages, phagocytic ability of mononuclear phagocyte cells dramatically increases [18]. For example, we observed an increase from 8.3% to 32.4% in phagocytosis of apoptotic neutrophils by differentiated macrophages compared to monocytes [19]. Using a self-designed apopto-phagocyte gene-array (***Table S1***) we examined expression changes during differentiation to explain this phenomenon. ***Table 1*** lists genes that were significantly up- or down-regulated during differentiation. The expression level of 29 genes of the analyzed 95 genes increased significantly and only 6 genes were down-regulated (e. g. C1QR1, ICAM3, PTX3, FPRL1) in accordance with our assumption that phagocytosis increase during macrophage differentiation is resulted from the induction of apopto - phagocytic genes. The expression level of MERTK in 2 of 3 donors was strongly up-regulated, though this did not prove to be significant. There was also a heterogenous group of genes with high level of expression in monocytes that did not change during differentiation: TGFB1, PECAM1, CD14, THBS1, CAPN 1 and 2, RHOG, ANXA1, PYCARD, ITGAX.

**Table 1:** Significant changes in the expression of apopto-phagocytic genes during macrophage differentiation (n = 3, p<0.05); down-regulated: FC<0.67, slightly up-regulated: 1.67<FC<3.00, moderately up-regulated 3.00<FC<10.00; highly up-regulated FC>10.00.

HUGO name*	Gene description	<FC>
***Down-regulated genes***		
ICAM3	intercellular adhesion molecule 3	0.06
CASP5	caspase 5, apoptosis-related cysteine protease	0.09
FPRL1	formyl peptide receptor-like 1	0.12
C1QR1	complement component 1, q subcomponent, receptor 1	0.13
ALOX5	arachidonate 5-lipoxygenase	0.14
PTX3	pentaxin-related gene, rapidly induced by IL-1 beta	0.24
***Slightly up-regulated genes***		
ITGB2	integrin, beta 2 (antigen CD18 (p95))	1.69
APG16L	autophagy protein 16-like	1.97
BIRC1	Baculoviral IAP repeat-containing protein 1	2.00
IRF8	interferon regulatory factor 8	2.00
DNASE1	deoxyribonuclease II, lysosomal	2.19
IL4R	interleukin 4 receptor	2.39
ANXA5	annexin A5	2.52
APG5L	autophagy protein 5-like	2.67
AXL	AXL receptor tyrosine kinase	2.82
***Moderately up-regulated genes***		
CRK	CDNA FLJ38130 fis, clone D6OST2000464	3.47
PTPNS1	protein tyrosine phosphatase, non-receptor type substrate 1	3.58
CARD4	caspase recruitment domain family, member 4	3.59
LRP1	low density lipoprotein-related protein 1 (alpha-2-macroglobulin receptor)	3.75
IL10	interleukin 10	4.38
ABCA1	ATP-binding cassette, sub-family A (ABC1), member 1	5.39
CALR	calreticulin	5.67
GAS6	growth arrest-specific 6	5.72
CD68	CD68 antigen	5.94
IL18	interleukin 18 (interferon-gamma-inducing factor)	6.42
DNASE2	deoxyribonuclease I	6.60
SCARB1	scavenger receptor class B, member 1	6.63
***Highly up-regulated genes***		
FCGR2B	Fc fragment of IgG, low affinity IIb, receptor for (CD32)	12.25
ADORA3	adenosine A3 receptor	22.09
DOCK1	dedicator of cytokinesis 1	23.31
TGM2	tissue transglutaminase-2	23.45
C1QA	complement component 1, q subcomponent, alpha polypeptide	39.24
MSR1	macrophage scavenger receptor 1	68.21
PPARG	peroxisome proliferative activated receptor, gamma	81.47
ITGB5	integrin, beta 5	178.52

*Gene Symbol defined by HUGO Gene Nomenclature Committee (HGNC, http://www.genenames.org), FC: Fold change.

### Comparison of gene expression patterns of macrophages from different donors

**Figure 1:**
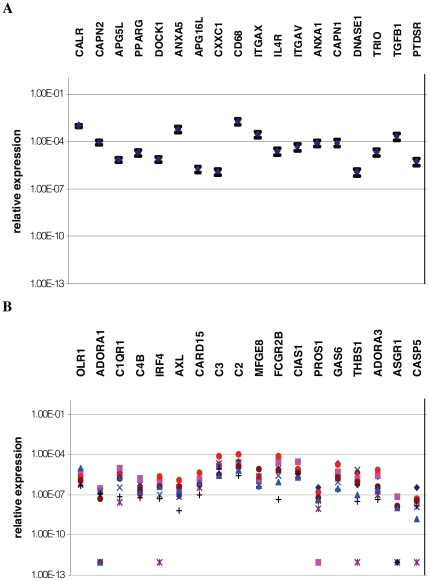
Relative levels of gene expression in macrophages differentiated from monocytes of different donors. A: non-variable genes (genes with SD below 20 percentile) represented by average values and error, B: variable genes (genes with SD over 80 percentile) represented by individual dots, n = 8.

The relative expressions of apopto-phagocytic genes in macrophages differentiated from monocytes of different donors were also compared. We observed that there were non-variable genes, whose relative expression level varied only 20–50% amongst donors, and there were highly variable ones, whose expression level varied in 2–3 orders of magnitude (Figure 1). The expression level of the non-variable genes was typically higher and some of them were up-regulated during differentiation (e.g. PPARγ, DOCK1, CD68, ITGAX etc.). Based on these data it can be assumed that these genes encode proteins that are essential to macrophage functions and most of them are induced during differentiation. Phagocytic capacity of each of the eight different donors was measured but no correlation was found between gene expression levels and phagocytic capacity (Table S2).

### Effect of dexamethasone on the gene expression pattern of differentiated macrophages

**Figure 2:**
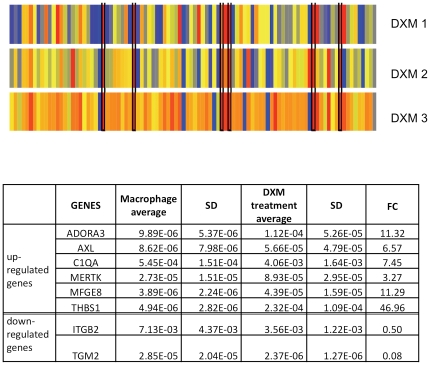
Changes in normalized expression levels of apopto-phagocytic genes in macrophages as a result of DXM tretament. Macrophages were differentiated in the presence or absence of dexamethasone. Relative gene expression data of DXM treated macrophages were normalized to non-treated ones and are shown in heat map. Red: up-regulation, blue: down-regulation. Six genes were up- and 2 down-regulated as demonstrated by the heat map; the ones up-regulated in each donor (n = 3, p<0.05) are highlighted. Table shows average relative expression levels of control and DXM treated macrophages, their standard deviation (SD) and fold change (FC) as a ratio of expression levels of DXM treated and control macrophages.

Glucocorticoids can highly increase the phagocytic capacity of macrophages toward apoptotic neutrophils [12], [13]; according to our data obtained with macrophages from a large number of donors and carrying out differentiation for 5 days in the presence of 1 µM DXM there is usually a 2–2.5 fold enhancement of phagocytosis (n = 15, data not shown). As a result of DXM treatment 6 apopto-phagocytic genes were significantly up-regulated and 2 down-regulated as compared to macrophages differentiated in the absence of DXM (Figure 2.). The DXM induced genes encode either receptors (MERTK, AXL, ADORA3) or bridge-building proteins (C1QA, MFGE8, THBS1) between phagocytes and apoptotic cell. It should be noted that 4 of them (AXL, ADORA3, MFGE8 and THBS1) were from the group of genes whose expression was quite variable among donors, and the other 4 of them (MERTK, C1QA, ITGB2, TGM2) were in the intermediate group regarding their variance.

### Functional effect of knocking-down of DXM up-regulated genes on phagocytosis

**Figure 3:**
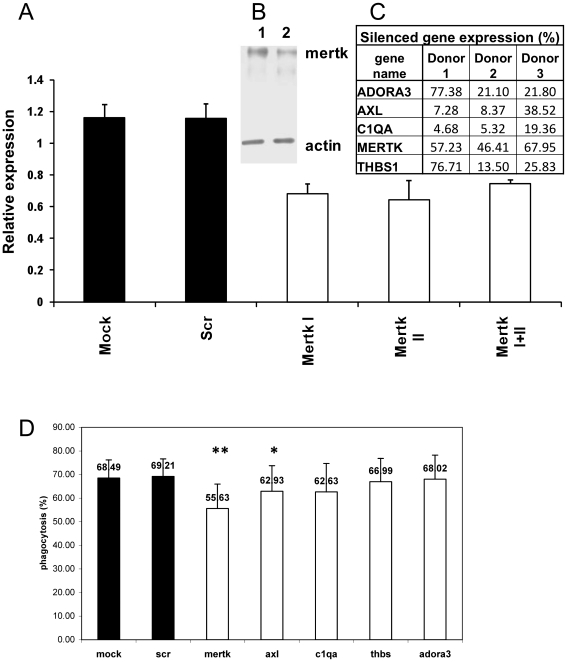
Silencing of DXM up-regulated genes and its effect on the phagocytosis of apoptotic cells. A, B, C: Knock-down effect in human macrophages using electroporation (siRNS cc. 3 µM) on mRNA (A, C) and protein level (B). A: MERTK I, MERTK II, MERTK I+II: MERTK knock-downs silenced by MERTK siRNA I, II or both; B: Western blot analysis of MERT expression (1: Mock, 2: MERTK KD); C: gene expression level of DXM up-regulated genes after silencing in 3 different donors (in per cent as compared to mock). D: Phagocytosis of apoptotic cells by macrophages after knocking down DXM up-regulated genes. scr: scrambled siRNA, Mertk I+II: double knock-down of Mertk I and II siRNAs, Mertk: Mertk I siRNA. ** p<0.01, n = 4; * p<0.1, n = 3.

To investigate whether DXM induced up-regulation has a direct effect on the enhanced phagocytic capacity of macrophages the up-regulated genes were silenced using siRNA. The silencing efficiency varied in the different genes (Figure 3C). Although the weakest silencing effect was achieved for MERTK (about 50% knock-down effect, Figure 3A, C) the phagocytosis of only the MERTK knock down, DXM treated macrophages decreased significantly (Figure 3D). The combination of MERTK silencing with siRNAs for AXL or any of the other DXM up-regulated genes as well as bridging molecules with each other did not show a synergistic effect (data not shown).

### Impact of blocking Mertk receptor on the phagocytosis of apoptotic neutrophils

**Figure 4:**
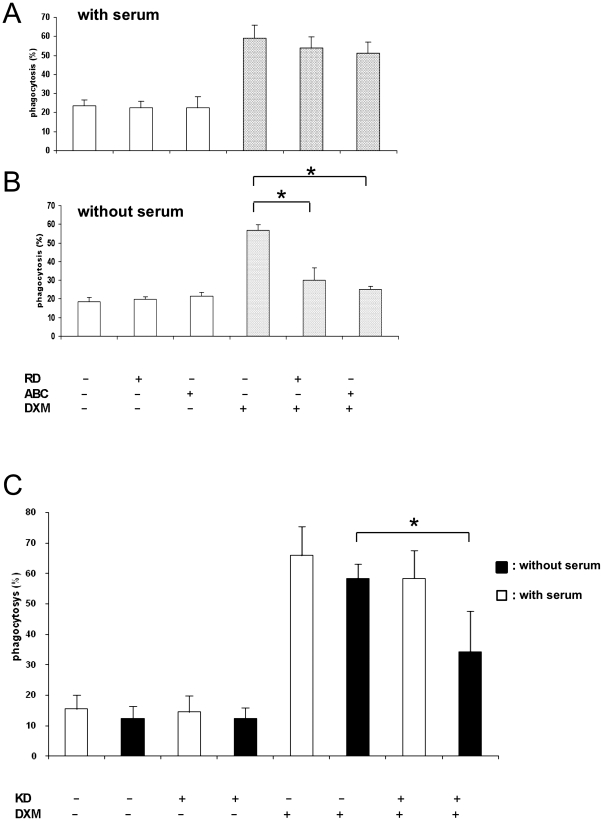
Effect of blocking cell surface Mertk by antibodies on the phagocytosis of apoptotic neutrophils. Mertk on the surface of DXM treated and nontreated macrophages was blocked by different antibodies in the presence (A) and in the absence (B) of serum; spotted bar: DXM treated, empty bar: DXM non-treated C: Serum effect on the phagocytosis of apoptotic neutrophils by Mertk knock-down cells. RD and ABC indicate antibodies of R&D Systems and Abcam, respectively. KD: Mertk knock down silenced by Mertk II siRNA; filled bar: including serum, empty bar: excluding serum. * p<0.05, n = 3.

We also investigated whether blocking of Mertk with an antibody verifies its key role in DXM induced increase of phagocytosis. It was found that while Mertk blocking antibodies did not inhibit phagocytosis by macrophages differentiated in the absence of DXM, the DXM-induced increase of phagocytic capacity of macrophages could be prevented by treatment with this antibody (Figure 4 A, B). This effect was much weaker when the assay was performed in the presence of serum, when the enhancing effect of DXM treatment on macrophages was also apparent, suggesting that the consequence of blocking Mertk can be compensated by other engulfing mechanisms in macrophages.

In order to exclude the possible role of soluble Mer in serum that inhibits macrophage clearance of apoptotic cells [35] we repeated this experiment using Mertk knock-downs cells instead of blocking antibodies (Figure 4 C). It was found that knocking down Mertk expression resulted in more effective blocking of DXM-induced increase in phagocytosis of apoptotic cells in the absence of serum. This demonstrates the existence of a Mertk independent DXM mediated phagocytic process operating in the presence of serum.

### Expression of DXM induced genes in THP-1 cells

**Figure 5:**
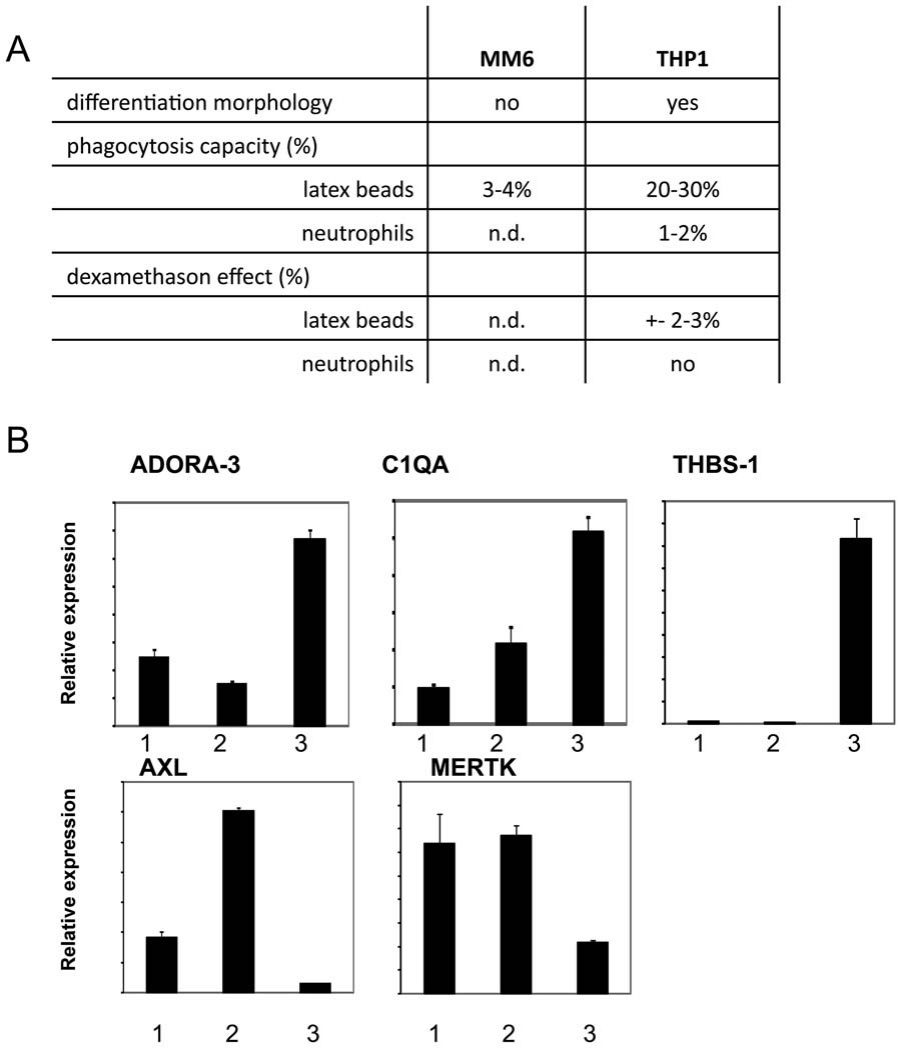
Different expression pattern of DXM-regulated genes in differentiated THP-1 cells compared to human macrophages. A: Phagocytosis capacity of DXM treated and non-treated human monocyte-macrophage like cell lines are show. n.d.: not detectable; B: Expression level of DXM-regulated genes in differentiated THP-1 cells. B: 1. PMA, 2 PMA+1,25-dihydroxyvitamin, 3. PMA+1,25-dihydroxyvitamin+ DXM treated THP-1 cells.

To gain further information about the correlation between DXM mediated gene induction in macrophages and their phagocytosis capacity toward apoptotic neutrophils, the effect of DXM treatment on human macrophage like cell lines was also investigated. Mono Mac 6 cells exhibit many characteristics of mature blood monocytes [27] but we found that their phagocytic capacity toward apoptotic neutrophils or carboxylate-modified latex beads (which act as surrogate apoptotic cells) was very low (Figure 5A). Human THP- 1 leukemia cells are known to differentiate along the monocytic lineage following exposure to phorbol-12-myristate-13-acetate (PMA) or 1,25-dihydroxyvitamin D3 (VD3) [20]. Although THP-1 cells are capable of taking up carboxylated polystyrene latex beads, they can engulf apoptotic neutrophils very poorly and DXM treatment could not enhance the phagocytosis of either latex beads or apoptotic cells. Checking the expression of those genes which were up-regulated in human macrophages by DXM treatment we found that although ADORA3, C1QA and THBS1 were induced, the expression level of both MERTK and AXL (the two tyrosine kinase genes) dropped in contrast to HMDMs (Figure 5B).

## Discussion

Differentiation of monocytes to macrophages is associated with an enhancement of phagocytosis of apoptotic cells and glucocorticoids are capable to further augment this process [19], [22]. In this study we have examined the gene expression changes that underlie these enhancements in phagocytic capacity. We used a TaqMan Low Density Array configuration containing TaqMan assays for the apopto-phagocytic genes of the following categories: receptors (integrins, scavenger receptors, adenosine receptors, tyrosine kinases, etc.), bridging molecules, signal generators, effector, cytokines, nuclear receptors, engulfment genes, autophagy genes, interferon regulatory family genes (Table S1.). We found that the expression level of the majority of these genes were altered and most of them up-regulated during differentiation of monocytes to macrophages. Among the highly and moderately up-regulated genes there are several genes whose importance in the phagocytosis of macrophages have already been described either as receptors (scavenger receptors as CD36, CD68 and CD 204, FcγRIIB, integrin b5), as bridging molecules (C1QA, GAS6) [23], [24], as a molecule with functions on both apoptotic and engulfing cells (calreticulin), or as effector molecules participating in different signaling pathways e.g. CRK, PTPNS1, CARD4, ADORA3, DOCK1 and TGM2 [31], [32]). In a recent paper we demonstrated that during differentiation of macrophages natural ligands of PPARg (also highly up-regulated during differentiation) are formed, regulating the expression of genes responsible for effective clearance of apoptotic cells and macrophage-mediated inflammatory responses [19]. The reason of the down-regulation of genes with previously demonstrated functions in different phagocytosis pathways (C1QR1, ICAM3, PTX3, FPRL1) needs to be clarified in future studies.

The variability of expression levels of apopto-phagocytic genes among differentiated macrophages of human donors was also investigated and striking differences were found. Although there were several genes with very low variability of SD between 20–50%, the expression levels of another set of genes varied in 2–3 orders of magnitude. The average relative expression level of non-variable genes was typically higher than that of the variable ones. During macrophage differentiation up-regulated genes partly overlapped with non-variable genes in some cases such as calreticulin, PPARγ and CD68 while some with variable ones like FcγRIIB, GAS6, ADORA3. The non-variable gene set might represent permanently switched on tools for the phagocytosis machinery while the variable set may provide alternative ways for phagocytosis under various local conditions to which macrophages may be exposed.

Glucocorticoids are still the most potent immunosuppressive agents with complex and cell type specific actions on immune cells [28]. Macrophages are relevant targets for anti-inflammatory therapy by glucocorticoids not only because of their large repertoire of inflammatory regulators, but also their alternative anti-inflammatory phenotype that was described recently [15]. It was found that GCs modulate the expression of 130 genes, including anti-inflammatory ones and those involved in chemotaxis, phagocytosis and antioxidative stress, while suppresses pro-inflammatory genes related to apoptosis, adhesion and T-cell chemotaxis. However, the authors generated the expression profiles of monocytes following only a 18 h stimulation with fluticasone, a new generation GC with a higher binding affinity to the GR than dexamethasone; this is a short time course and GCs may modulate gene transcription differently over a longer time period [29]. According to our results most of the up-regulated apopto-phagocytic genes after 5 days of DXM exposure (C1QA, MFGE8, THBS1, ADORA3, MFGE8) are the same as those observed after 18 hours treatment. However, several genes that were found to be induced after 18 h stimulation were not responding after 5 days treatment; for example, the receptor for anti-inflammatory mediators FPR1 was not up-regulated or the adhesion molecules ITGAL, CD36 or OLR1 were not down-regulated after the long term DXM treatment. This means that in macrophages only part of the apopto-phagocytic genes is regulated on the long term by glucocorticoids as compared to the early or transient gene expression pattern.

Mertk is a member of the TAM (TYRO3, AXL, MER) receptor protein tyrosine kinase family which have substantial roles in innate immunity. Their known two ligands, growth-arrest-specific 6 (GAS6) and protein S bind to and activate all the three TAM receptors with different affinities and due to their Gla domains they also can bind phosphatidylserine on apoptotic cells simultaneously [30]. However, only Mertk seems to be critical for the clearance of apoptotic cells that was demonstrated using mer^kd^ mice with a truncation of the cytoplasmic part of Mer, where macrophages from these mice were deficient in the clearance of apoptotic thymocytes [16]. This deficiency may result in autoimmunity that is treated still most efficiently by glucocorticoids. Our results showed that Mertk has a pivotal role in dexamethasone induced enhancement of phagocytosis. Using blocking antibodies we proved that masking Mertk has no effect on the phagocytic potential of untreated monocyte derived macrophages.

In a recent paper McColl et al. suggested that glucocorticoids regulate a switch from a serum independent to a serum-dependent apoptotic cell recognition mechanism, which can be recapitulated with protein S, the purified Mertk ligand, and involves macrophage Mertk [17]. In our study we could demonstrate that the inhibitory effect of blocking Mertk on DXM facilitated phagocytosis of apoptotic cells can be almost completely diminished if human autologous serum was present during phagocytosis. Furthermore, the enhancing effect of dexamethasone pre-treatment of macrophages on phagocytosis of apoptotic cells could be also observed in serum free conditions and blocking of Mertk has no significant effect in the presence, but it has in the absence of serum. This implies that the proposed glucocorticoid induced switch in the apoptotic cell recognition mechanisms [17] is not only influenced by the presence of serum and Mertk is not the only player in the switch as blocking it in the presence of serum had a vary moderate effect on the phagocytosis capacity of macrophages.

The discrepancy in the DXM effect in serum free conditions between McColl's and our data can be explained by the application of differently induced apoptotic neutrophils. We exclusively used neutrophils cultured in the presence of serum that mimics physiological processes occurring in the human body. The fact that phagocytosis of neutrophils made apoptotic in the presence of serum could be augmented by glucocorticoids, and this was efficiently inhibited in a Mertk dependent manner, underlines that Mertk has a pivotal role in this phagocytic process which is mainly serum independent. On the other hand, the enhancement effect of glucocorticoid treatment on phagocytosis have additional elements which becomes apparent only when serum is present during engulfment of apoptotic cells; the identification of these molecular pathways warrants further studies. Our data also confirms that phagocytes respond differently even to the same apoptotic cell types if they were induced in different circumstances.

The differentiation of monocytes into macrophages is a crucial step in the early immune response, and the human monocytic leukaemia cell line THP1 has been frequently used as an *in vitro* model to investigate this process. Several compounds such as phorbol 12-myristate 13-acetate (PMA), 1,25-dihydroxyvitamin D3, retinoic acid, or eukaryotic cytokines have been used to trigger this differentiation process, which can be monitored by changes in morphology, adherence or expression of surface markers [20]–[22]. Increase of phagocytosis also could be an important feature of differentiation; however, glucocorticoid elicited differentiation can induce only a very small effect on the phagocytosis of apoptotic neutrophils in THP1 cells and, in contrast to other apopto-phagocytic genes that were up-regulated in HMDMs, Mertk was down-regulated in THP1 cells. This also highlights the profound role of Mertk in glucocorticoid induced up-regulation of apoptotic cell phagocytosis, since its down-regulation in THP-1 cells explains why glucocorticoids can not augment phagocytosis of apoptotic neutrophils by these cells.

Efficient phagocytic clearance of apoptotic cells is crucial in many biological processes. If this machinery is impaired the apoptotic cell debris can contribute to the development of sterile inflammation and autoimmunity [25]. During the resolution phase of inflammation apoptotic neutrophils are cleared away by a rapid and efficient process that does not stimulate proinflammatory macrophage responses [26]. Glucocorticoids represent one of the most powerful clinical treatments for a range of inflammatory conditions including severe acute inflammation and autoimmune diseases. Understanding the mechanism of action used by glucocorticoids to enhance clearance of dying cells, to which this study has provided further details, may lead to the development of novel therapeutic compounds with higher specificity and fewer or less side effects.

## Supporting Information

Table S1 Gene list of the apopto-phagocyte panel. Gene symbol (HUGO), Alias, Gene description, HGNC code, and Location is given for each gene as defined by HUGO Gene Nomenclature Committee (HGNC, http://www.genenames.org).
(DOC)

Table S2 Correlation data between gene expression levels and phagocytic capacity. It contains HUGO gene ID and R2 calculated with linear regression by *Partial Least Squares (PLS)* for correlation between gene expression and phagocytosis data.
(DOC)
