# Supplementary material for: Differentiation and Glucocorticoid Regulated Apopto-Phagocytic Gene Expression Patterns in Human Macrophages. Role of Mertk in Enhanced Phagocytosis
Source: PLoS One. 2011 Jun 24;6(6):e21349. doi: 10.1371/journal.pone.0021349 (PMC3123306; doi:10.1371/journal.pone.0021349)
Supplement: Table S1 — Gene list of the apopto-phagocyte panel. Gene symbol (HUGO), Alias, Gene description, HGNC code, and Location is given for each gene as defined by HUGO Gene Nomenclature Committee (HGNC, http://www.genenames.org). (DOC) [file pone.0021349.s001.doc]

**Table S1:** Gene list of the apopto-phagocyte panel

| **Gene symbol (HUGO)** | **Alias** | **Gene description** | **HGNC code** | **Location** |
| --- | --- | --- | --- | --- |
| [ABCA1](http://www.genenames.org/data/hgnc_data.php?hgnc_id=29) | ABCA1 | ATP-binding cassette, sub-family A (ABC1), member 1 | HGNC:29 | 9q31 |
| [ADORA1](http://www.genenames.org/data/hgnc_data.php?hgnc_id=262) | ADORA1 | adenosine A1 receptor | HGNC:262 | 1q32.1 |
| [ADORA2A](http://www.genenames.org/data/hgnc_data.php?hgnc_id=263) | ADORA2A | adenosine A2a receptor | HGNC:263 | 22q11.23 |
| [ADORA3](http://www.genenames.org/data/hgnc_data.php?hgnc_id=268) | ADORA3 | adenosine A3 receptor | HGNC:268 | 1p21-p13 |
| [ALOX12](http://www.genenames.org/data/hgnc_data.php?hgnc_id=429) | ALOX12 | arachidonate 12-lipoxygenase | HGNC:429 | 17p13.1 |
| [ALOX5](http://www.genenames.org/data/hgnc_data.php?hgnc_id=435) | ALOX5 | arachidonate 5-lipoxygenase | HGNC:435 | 10q11.2 |
| [ANXA1](http://www.genenames.org/data/hgnc_data.php?hgnc_id=533) | ANXA1 | annexin A1 | HGNC:533 | 9q21.13 |
| [ANXA5](http://www.genenames.org/data/hgnc_data.php?hgnc_id=543) | ANXA5 | annexin A5 | HGNC:543 | 4q27 |
| [ATG12](http://www.genenames.org/data/hgnc_data.php?hgnc_id=588) | APG12L | ATG12 autophagy related 12 homolog (S. cerevisiae) | HGNC:588 | 5q21-q22 |
| [ATG16L1](http://www.genenames.org/data/hgnc_data.php?hgnc_id=21498) | APG16L | ATG16 autophagy related 16-like 1 (S. cerevisiae) | HGNC:21498 | 2q37.1 |
| [ATG5](http://www.genenames.org/data/hgnc_data.php?hgnc_id=589) | APG5L | ATG5 autophagy related 5 homolog (S. cerevisiae) | HGNC:589 | 6q21 |
| [APOH](http://www.genenames.org/data/hgnc_data.php?hgnc_id=616) | APOH | apolipoprotein H (beta-2-glycoprotein I) | HGNC:616 | 17q23-qter |
| [PYCARD](http://www.genenames.org/data/hgnc_data.php?hgnc_id=16608) | ASC | PYD and CARD domain containing | HGNC:16608 | 16p11.2 |
| [ASGR1](http://www.genenames.org/data/hgnc_data.php?hgnc_id=742) | ASGR1 | asialoglycoprotein receptor 1 | HGNC:742 | 17p13-p11 |
| [AXL](http://www.genenames.org/data/hgnc_data.php?hgnc_id=905) | AXL | AXL receptor tyrosine kinase | HGNC:905 | 19q13.1 |
| [BCAR1](http://www.genenames.org/data/hgnc_data.php?hgnc_id=971) | BCAR1 | breast cancer anti-estrogen resistance 1 | HGNC:971 | 16q22-q23 |
| [BECN1](http://www.genenames.org/data/hgnc_data.php?hgnc_id=1034) | BECN1 | beclin 1, autophagy related | HGNC:1034 | 17q21 |
| [NAIP](http://www.genenames.org/data/hgnc_data.php?hgnc_id=7634) | BIRC1 | NLR family, apoptosis inhibitory protein | HGNC:7634 | 5q13.2 |
| [C1QA](http://www.genenames.org/data/hgnc_data.php?hgnc_id=1241) | C1QA | complement component 1, q subcomponent, A chain | HGNC:1241 | 1p36.3-p34.1 |
| [CD93](http://www.genenames.org/data/hgnc_data.php?hgnc_id=15855) | C1QR1 | CD93 molecule | HGNC:15855 | 20p11.21 |
| [C2](http://www.genenames.org/data/hgnc_data.php?hgnc_id=1248) | C2 | complement component 2 | HGNC:1248 | 6p21.3 |
| [C3](http://www.genenames.org/data/hgnc_data.php?hgnc_id=1318) | C3 | complement component 3 | HGNC:1318 | 19p13.3-p13.2 |
| [C4A](http://www.genenames.org/data/hgnc_data.php?hgnc_id=1323) | C4A | complement component 4A (Rodgers blood group) | HGNC:1323 | 6p21.3 |
| [CALR](http://www.genenames.org/data/hgnc_data.php?hgnc_id=1455) | CALR | calreticulin | HGNC:1455 | 19p13.3-p13.2 |
| [CAPN1](http://www.genenames.org/data/hgnc_data.php?hgnc_id=1476) | CAPN1 | calpain 1, (mu/I) large subunit | HGNC:1476 | 11q13 |
| [CAPN2](http://www.genenames.org/data/hgnc_data.php?hgnc_id=1479) | CAPN2 | calpain 2, (m/II) large subunit | HGNC:1479 | 1q41-q42 |
| [NOD2](http://www.genenames.org/data/hgnc_data.php?hgnc_id=5331) | CARD15 | nucleotide-binding oligomerization domain containing 2 | HGNC:5331 | 16q12 |
| [NOD1](http://www.genenames.org/data/hgnc_data.php?hgnc_id=16390) | CARD4 | nucleotide-binding oligomerization domain containing 1 | HGNC:16390 | 7p15-p14 |
| [CASP1](http://www.genenames.org/data/hgnc_data.php?hgnc_id=1499) | CASP1 | caspase 1, apoptosis-related cysteine peptidase (interleukin 1, beta, convertase) | HGNC:1499 | 11q23 |
| [CASP5](http://www.genenames.org/data/hgnc_data.php?hgnc_id=1506) | CASP5 | caspase 5, apoptosis-related cysteine peptidase | HGNC:1506 | 11q22.2-q22.3 |
| [CD14](http://www.genenames.org/data/hgnc_data.php?hgnc_id=1628) | CD14 | CD14 molecule | HGNC:1628 | 5q22-q32 |
| [CD47](http://www.genenames.org/data/hgnc_data.php?hgnc_id=1682) | CD47 | CD47 molecule | HGNC:1682 | 3q13.1-q13.2 |
| [CD68](http://www.genenames.org/data/hgnc_data.php?hgnc_id=1693) | CD68 | CD68 molecule | HGNC:1693 | 17p13 |
| [NLRP3](http://www.genenames.org/data/hgnc_data.php?hgnc_id=16400) | CIAS1 | NLR family, pyrin domain containing 3 | HGNC:16400 | 1q44 |
| [CRK](http://www.genenames.org/data/hgnc_data.php?hgnc_id=2362) | CRK | v-crk sarcoma virus CT10 oncogene homolog (avian) | HGNC:2362 | 17p13 |
| [CRP](http://www.genenames.org/data/hgnc_data.php?hgnc_id=2367) | CRP | C-reactive protein, pentraxin-related | HGNC:2367 | 1q21-q23 |
| [CXXC1](http://www.genenames.org/data/hgnc_data.php?hgnc_id=24343) | CXXC1 | CXXC finger 1 (PHD domain) | HGNC:24343 | 18q12 |
| [DNASE1](http://www.genenames.org/data/hgnc_data.php?hgnc_id=2956) | DNASE1 | deoxyribonuclease I | HGNC:2956 | 16p13.3 |
| [DNASE2](http://www.genenames.org/data/hgnc_data.php?hgnc_id=2960) | DNASE2 | deoxyribonuclease II, lysosomal | HGNC:2960 | 19p13.2-q13.2 |
| [DOCK1](http://www.genenames.org/data/hgnc_data.php?hgnc_id=2987) | DOCK1 | dedicator of cytokinesis 1 | HGNC:2987 | 10q26.13-q26.3 |
| [EDIL3](http://www.genenames.org/data/hgnc_data.php?hgnc_id=3173) | EDIL3 | EGF-like repeats and discoidin I-like domains 3 | HGNC:3173 | 5q14 |
| [ELMO1](http://www.genenames.org/data/hgnc_data.php?hgnc_id=16286) | ELMO1 | engulfment and cell motility 1 | HGNC:16286 | 7p14.1 |
| [ELMO2](http://www.genenames.org/data/hgnc_data.php?hgnc_id=17233) | ELMO2 | engulfment and cell motility 2 | HGNC:17233 | 20q13 |
| [FCGR2B](http://www.genenames.org/data/hgnc_data.php?hgnc_id=3618) | FCGR2B | Fc fragment of IgG, low affinity IIb, receptor (CD32) | HGNC:3618 | 1q23 |
| [FPR2](http://www.genenames.org/data/hgnc_data.php?hgnc_id=3827) | FPRL1 | formyl peptide receptor 2 | HGNC:3827 | 19q13.3-q13.4 |
| [GAS6](http://www.genenames.org/data/hgnc_data.php?hgnc_id=4168) | GAS6 | growth arrest-specific 6 | HGNC:4168 | 13q34 |
| [GRLF1](http://www.genenames.org/data/hgnc_data.php?hgnc_id=4591) | GRLF1 | glucocorticoid receptor DNA binding factor 1 | HGNC:4591 | 19q13.32 |
| [GULP1](http://www.genenames.org/data/hgnc_data.php?hgnc_id=18649) | GULP1 | GULP, engulfment adaptor PTB domain containing 1 | HGNC:18649 | 2q32.3-q33 |
| [ICAM3](http://www.genenames.org/data/hgnc_data.php?hgnc_id=5346) | ICAM3 | intercellular adhesion molecule 3 | HGNC:5346 | 19p13.3-p13.2 |
| [IRF8](http://www.genenames.org/data/hgnc_data.php?hgnc_id=5358) | ICSBP1 | interferon regulatory factor 8 | HGNC:5358 | 16q24.1 |
| [IL10](http://www.genenames.org/data/hgnc_data.php?hgnc_id=5962) | IL10 | interleukin 10 | HGNC:5962 | 1q31-q32 |
| [IL12B](http://www.genenames.org/data/hgnc_data.php?hgnc_id=5970) | IL12B | interleukin 12B (natural killer cell stimulatory factor 2, cytotoxic lymphocyte maturation factor 2, p40) | HGNC:5970 | 5q31.1-q33.1 |
| [IL18](http://www.genenames.org/data/hgnc_data.php?hgnc_id=5986) | IL18 | interleukin 18 (interferon-gamma-inducing factor) | HGNC:5986 | 11q22.2-q22.3 |
| [IL23A](http://www.genenames.org/data/hgnc_data.php?hgnc_id=15488) | IL23A | interleukin 23, alpha subunit p19 | HGNC:15488 | 12q13.13 |
| [IL4R](http://www.genenames.org/data/hgnc_data.php?hgnc_id=6015) | IL4R | interleukin 4 receptor | HGNC:6015 | 16p12.1-p11.2 |
| [IL6](http://www.genenames.org/data/hgnc_data.php?hgnc_id=6018) | IL6 | interleukin 6 (interferon, beta 2) | HGNC:6018 | 7p21-p15 |
| [PNPLA8](http://www.genenames.org/data/hgnc_data.php?hgnc_id=28900) | IPLA2(GAMMA) | patatin-like phospholipase domain containing 8 | HGNC:28900 | 7q31 |
| [IRF1](http://www.genenames.org/data/hgnc_data.php?hgnc_id=6116) | IRF1 | interferon regulatory factor 1 | HGNC:6116 | 5q23-q31 |
| [IRF4](http://www.genenames.org/data/hgnc_data.php?hgnc_id=6119) | IRF4 | interferon regulatory factor 4 | HGNC:6119 | 6p25-p23 |
| [IRF5](http://www.genenames.org/data/hgnc_data.php?hgnc_id=6120) | IRF5 | interferon regulatory factor 5 | HGNC:6120 | 7q32 |
| [IRF7](http://www.genenames.org/data/hgnc_data.php?hgnc_id=6122) | IRF7 | interferon regulatory factor 7 | HGNC:6122 | 11p15.5 |
| [ITGAM](http://www.genenames.org/data/hgnc_data.php?hgnc_id=6149) | ITGAM | integrin, alpha M (complement component 3 receptor 3 subunit) | HGNC:6149 | 16p11.2 |
| [ITGAV](http://www.genenames.org/data/hgnc_data.php?hgnc_id=6150) | ITGAV | integrin, alpha V (vitronectin receptor, alpha polypeptide, antigen CD51) | HGNC:6150 | 2q31-q32 |
| [ITGAX](http://www.genenames.org/data/hgnc_data.php?hgnc_id=6152) | ITGAX | integrin, alpha X (complement component 3 receptor 4 subunit) | HGNC:6152 | 16p11.2 |
| [ITGB2](http://www.genenames.org/data/hgnc_data.php?hgnc_id=6155) | ITGB2 | integrin, beta 2 (complement component 3 receptor 3 and 4 subunit) | HGNC:6155 | 21q22.3 |
| [ITGB3](http://www.genenames.org/data/hgnc_data.php?hgnc_id=6156) | ITGB3 | integrin, beta 3 (platelet glycoprotein IIIa, antigen CD61) | HGNC:6156 | 17q21.32 |
| [ITGB5](http://www.genenames.org/data/hgnc_data.php?hgnc_id=6160) | ITGB5 | integrin, beta 5 | HGNC:6160 | 3q21.2 |
| [LRP1](http://www.genenames.org/data/hgnc_data.php?hgnc_id=6692) | LRP1 | low density lipoprotein receptor-related protein 1 | HGNC:6692 | 12q13-q14 |
| [MAP1LC3A](http://www.genenames.org/data/hgnc_data.php?hgnc_id=6838) | MAP1LC3A | microtubule-associated protein 1 light chain 3 alpha | HGNC:6838 | 20q11.22 |
| [MERTK](http://www.genenames.org/data/hgnc_data.php?hgnc_id=7027) | MERTK | c-mer proto-oncogene tyrosine kinase | HGNC:7027 | 2q14.1 |
| [MFGE8](http://www.genenames.org/data/hgnc_data.php?hgnc_id=7036) | MFGE8 | milk fat globule-EGF factor 8 protein | HGNC:7036 | 15q25 |
| [MSR1](http://www.genenames.org/data/hgnc_data.php?hgnc_id=7376) | MSR1 | macrophage scavenger receptor 1 | HGNC:7376 | 8p22 |
| [NLRP12](http://www.genenames.org/data/hgnc_data.php?hgnc_id=22938) | NALP12 | NLR family, pyrin domain containing 12 | HGNC:22938 | 19q13.42 |
| [NFKB1](http://www.genenames.org/data/hgnc_data.php?hgnc_id=7794) | NFKB1 | nuclear factor of kappa light polypeptide gene enhancer in B-cells 1 | HGNC:7794 | 4q24 |
| [OLR1](http://www.genenames.org/data/hgnc_data.php?hgnc_id=8133) | OLR1 | oxidized low density lipoprotein (lectin-like) receptor 1 | HGNC:8133 | 12p13.1-p12.3 |
| [PECAM1](http://www.genenames.org/data/hgnc_data.php?hgnc_id=8823) | PECAM1 | platelet/endothelial cell adhesion molecule | HGNC:8823 | 17q23.3 |
| [PPARG](http://www.genenames.org/data/hgnc_data.php?hgnc_id=9236) | PPARG | peroxisome proliferator-activated receptor gamma | HGNC:9236 | 3p25 |
| [PROS1](http://www.genenames.org/data/hgnc_data.php?hgnc_id=9456) | PROS1 | protein S (alpha) | HGNC:9456 | 3p11-q11.2 |
| [PTAFR](http://www.genenames.org/data/hgnc_data.php?hgnc_id=9582) | PTAFR | platelet-activating factor receptor | HGNC:9582 | 1p35-p34.3 |
| [JMJD6](http://www.genenames.org/data/hgnc_data.php?hgnc_id=19355) | PTDSR | jumonji domain containing 6 | HGNC:19355 | 17q25 |
| [PTGER2](http://www.genenames.org/data/hgnc_data.php?hgnc_id=9594) | PTGER2 | prostaglandin E receptor 2 (subtype EP2), 53kDa | HGNC:9594 | 14q22 |
| [PTK2](http://www.genenames.org/data/hgnc_data.php?hgnc_id=9611) | PTK2 | PTK2 protein tyrosine kinase 2 | HGNC:9611 | 8q24.3 |
| [SIRPA](http://www.genenames.org/data/hgnc_data.php?hgnc_id=9662) | PTPNS1 | signal-regulatory protein alpha | HGNC:9662 | 20p13 |
| [PTX3](http://www.genenames.org/data/hgnc_data.php?hgnc_id=9692) | PTX3 | pentraxin 3, long | HGNC:9692 | 3q25 |
| [RAC1](http://www.genenames.org/data/hgnc_data.php?hgnc_id=9801) | RAC1 | ras-related C3 botulinum toxin substrate 1 (rho family, small GTP binding protein Rac1) | HGNC:9801 | 7p22 |
| [RAP1A](http://www.genenames.org/data/hgnc_data.php?hgnc_id=9855) | RAP1A | RAP1A, member of RAS oncogene family | HGNC:9855 | 1p13.3 |
| [RHOG](http://www.genenames.org/data/hgnc_data.php?hgnc_id=672) | RHOG | ras homolog gene family, member G (rho G) | HGNC:672 | 11p15.5-p15.4 |
| [SCARB1](http://www.genenames.org/data/hgnc_data.php?hgnc_id=1664) | SCARB1 | scavenger receptor class B, member 1 | HGNC:1664 | 12q24.32 |
| [TGFB1](http://www.genenames.org/data/hgnc_data.php?hgnc_id=11766) | TGFB1 | transforming growth factor, beta 1 | HGNC:11766 | 19q13.1 |
| [TGFBR1](http://www.genenames.org/data/hgnc_data.php?hgnc_id=11772) | TGFBR1 | transforming growth factor, beta receptor 1 | HGNC:11772 | 9q22 |
| [TGM2](http://www.genenames.org/data/hgnc_data.php?hgnc_id=11778) | TGM2 | transglutaminase 2 (C polypeptide, protein-glutamine-gamma-glutamyltransferase) | HGNC:11778 | 20q12 |
| [THBS1](http://www.genenames.org/data/hgnc_data.php?hgnc_id=11785) | THBS1 | thrombospondin 1 | HGNC:11785 | 15q15 |
| [TNF](http://www.genenames.org/data/hgnc_data.php?hgnc_id=11892) | TNF | tumor necrosis factor | HGNC:11892 | 6p21.3 |
| [TRIO](http://www.genenames.org/data/hgnc_data.php?hgnc_id=12303) | TRIO | triple functional domain (PTPRF interacting) | HGNC:12303 | 5p14-p15.1 |
| [TYRO3](http://www.genenames.org/data/hgnc_data.php?hgnc_id=12446) | TYRO3 | TYRO3 protein tyrosine kinase | HGNC:12446 | 15q15.1-q21.1 |
