# Supplementary material for: Differentiation and Glucocorticoid Regulated Apopto-Phagocytic Gene Expression Patterns in Human Macrophages. Role of Mertk in Enhanced Phagocytosis
Source: PLoS One. 2011 Jun 24;6(6):e21349. doi: 10.1371/journal.pone.0021349 (PMC3123306; doi:10.1371/journal.pone.0021349)
Supplement: Table S2 — Correlation data between gene expression levels and phagocytic capacity. It contains HUGO gene ID and R2 calculated with linear regression by Partial Least Squares (PLS) for correlation between gene expression and phagocytosis data. (DOC) [file pone.0021349.s002.doc]

**Table S2:** Correlation data between gene expression levels and phagocytic capacity

| **gene ID** | **R2** |  | **gene ID** | **R2** |
| --- | --- | --- | --- | --- |
| CAPN1 | 0.910304 |  | MFGE8 | 0.303817 |
| DOCK1 | 0.869375 |  | LRP1 | 0.303378 |
| ELMO2 | 0.854606 |  | MAP1LC3A | 0.300906 |
| ITGAX | 0.765719 |  | ADORA3 | 0.299850 |
| IRF7 | 0.761247 |  | CD14 | 0.292134 |
| ELMO1 | 0.748011 |  | CD68 | 0.287775 |
| IRF1 | 0.741918 |  | BECN1 | 0.279330 |
| TGFBR1 | 0.740491 |  | OLR1 | 0.269461 |
| CIAS1 | 0.679694 |  | MERTK | 0.241114 |
| THBS1 | 0.618305 |  | FPRL1 | 0.223887 |
| ITGB5 | 0.577376 |  | CASP1 | 0.210480 |
| APG16L | 0.564574 |  | ITGAM | 0.210431 |
| RHOG | 0.556974 |  | IPLA2(GAMMA) | 0.204479 |
| TGM2 | 0.550844 |  | DNASE2 | 0.203194 |
| PECAM1 | 0.538832 |  | CASP5 | 0.197212 |
| ALOX5 | 0.533738 |  | MSR1 | 0.192748 |
| AXL | 0.533518 |  | RAC1 | 0.180141 |
| PTGER2 | 0.522453 |  | SCARB1 | 0.179975 |
| PTPNS1 | 0.518732 |  | TGFB1 | 0.176682 |
| PTK2 | 0.471841 |  | PYCARD | 0.174080 |
| BIRC1 | 0.462539 |  | IRF5 | 0.171747 |
| PTDSR | 0.456496 |  | CARD15 | 0.164809 |
| ITGB2 | 0.455125 |  | TNF | 0.164106 |
| ITGAV | 0.452922 |  | NFKB1 | 0.151482 |
| ADORA2A | 0.448718 |  | C2 | 0.150705 |
| IL18 | 0.446768 |  | CRK | 0.137506 |
| ANXA5 | 0.446452 |  | IL6 | 0.124426 |
| IL4R | 0.440838 |  | C3 | 0.107094 |
| CARD4 | 0.439247 |  | DNASE1 | 0.102245 |
| GRLF1 | 0.397211 |  | ADORA1 | 0.098793 |
| BCAR1 | 0.394100 |  | FCGR2B | 0.090355 |
| PROS1 | 0.389507 |  | IL23A | 0.086128 |
| ICAM3 | 0.366308 |  | ASGR1 | 0.083692 |
| IL10 | 0.355555 |  | PTAFR | 0.081500 |
| C4B | 0.348625 |  | ABCA1 | 0.079742 |
| PPARG | 0.345407 |  | NALP12 | 0.070109 |
| CAPN2 | 0.337610 |  | IRF8 | 0.048294 |
| CALR | 0.335892 |  | IRF4 | 0.026283 |
| ITGB3 | 0.335656 |  | PTX3 | 0.010656 |
| C1QA | 0.334172 |  | TYRO3 | 0.000337 |
| APG12L | 0.327720 |  | GAS6 | 0.000008 |
| CXXC1 | 0.322565 |  | ALOX12 | 0 |
| APG5L | 0.311770 |  | APOH | 0 |
| ANXA1 | 0.311327 |  | CRP | 0 |
| C1QR1 | 0.307499 |  | EDIL3 | 0 |
| CD47 | 0.305481 |  | GULP1 | 0 |
| TRIO | 0.304243 |  | IL12B | 0 |
